# Unraveling the role of asymmetric excitatory and inhibitory synaptic inputs to retinal ganglion cell direction selectivity

**DOI:** 10.3389/fnins.2026.1816252

**Published:** 2026-06-03

**Authors:** Jin Y. Huang, W. Rowland Taylor, Dario A. Protti

**Affiliations:** 1 Education Innovation Theme, School of Medical Sciences, The University of Sydney, Camperdown, NSW, Australia; 2 School of Optometry and Helen Wills Neuroscience Institute, University of California, Berkeley, Berkeley, CA, United States; 3 Herbert Wertheim School of Optometry and Vision Science, The University of California, Berkeley, Berkeley, CA, United States; 4 Neuroscience Theme, School of Medical Sciences, The University of Sydney, Camperdown, NSW, Australia

**Keywords:** direction selectivity, dynamic-clamp recordings, excitatory conductance, inhibitory conductance, retinal ganglion cells, visual neuroscience

## Abstract

**Introduction:**

Understanding the mechanisms underlying direction selectivity in retinal ganglion cells (RGCs) is crucial in visual neuroscience. Retinal direction selectivity is critical for gaze stabilization through optokinetic and vestibulo-ocular reflexes, and its loss impairs the ability to stabilize gaze and track moving objects, potentially impacting behaviors that rely on accurate motion detection. The prevailing hypothesis proposes that the interplay between excitatory and inhibitory inputs is pivotal for the emergence of direction selectivity in RGCs.

**Methods:**

To dissect the contributions of these inputs, we employed dynamic-clamp recordings utilizing computationally modeled, synthetic excitatory and inhibitory conductances of varying amplitudes and time onsets in mouse RGCs.

**Results:**

The use of combinations of excitatory and inhibitory conductances with altered amplitude or timing in configurations not found in natural physiological conditions, allowed us to evaluate the specific contribution and impact of these modified components on direction selectivity. We found that asymmetries in both excitatory and inhibitory inputs are critical for the emergence of sharp directional tuning.

**Discussion:**

Our findings contribute to advance our understanding of the cellular and synaptic mechanisms that underlie retinal direction selectivity.

## 1 Introduction

The ability of the visual system to encode motion information is crucial for animals to navigate their environment, track preys and evade predators among other tasks (Matsumoto and Yonehara, 2023; Vaney et al., 2012; Wei, 2018). Processing of visual motion starts in the retina with direction selective ganglion cells (DSGCs) that respond strongly to stimuli moving in a preferred direction and weakly, or not at all, to stimuli moving in the opposite or null direction (Barlow and Hill, 1963; Barlow and Levick, 1965). Additionally, DSGCs respond with a gradient of responses of intermediate strength to stimuli moving in directions between these cardinal directions (Barlow and Levick, 1965; Taylor and Vaney, 2002; see Matsumoto and Yonehara, 2023 for recent review).

Over the past two decades, extensive research pinpointed at the spatial and temporal integration of excitatory and inhibitory inputs as the key mechanisms underlying the emergence of direction selectivity in retinal ganglion cells (RGCs). Taylor and Vaney (2002) described the fundamental synaptic mechanisms that confer direction selectivity to ON-OFF DSGCs, as consisting of presynaptic mechanisms that enhance excitation in the preferred direction and augment inhibition in the null direction. Moreover, they reported postsynaptic interactions involving excitation and spatially offset inhibition. Excitatory inputs to ON-OFF DSGCs primarily involve glutamate release from bipolar cells and acetylcholine release from starburst amacrine cells (SACs). Whilst stronger excitation in the preferred direction was confirmed by subsequent studies (Fried et al., 2002; Weng et al., 2005), further research unveiled additional complexities within these circuits and introduced potential inconsistencies that challenged previous understandings. Lee et al. (2010) found that the maximum cholinergic response amplitude in DSGCs, elicited by SAC stimulation, remained consistent irrespective of whether the presynaptic SACs were located on the preferred, null, or intermediate side. Direct measurements of glutamate release onto ON-OFF DSGC dendrites using the glutamate biosensor iGluSnFR reported a lack of directionally tuned excitation (Park et al., 2014). Similarly, measurements of calcium influx in the terminals of cone bipolar cells type 5 and 7, which provide inputs to ON-OFF DCGCs, found no evidence of direction selectivity in bipolar cell signaling in response to moving bars (Chen et al., 2014).

Furthermore, computational simulations of somatic recordings from morphologically realistic neurons highlighted a significant distortion of excitatory conductances recorded in voltage-clamp mode due to unclamped inhibitory conductances (Percival et al., 2019; Poleg-Polsky and Diamond, 2011). Although it was initially suggested that this distortion might lead to an underestimation of the magnitude of excitation measured during null direction motion, the weak correlation found between the magnitude of the directional inhibitory and excitatory signals in small DSGCs suggests that the directional tuning of excitatory inputs cannot be solely attributed to voltage clamp artifacts (Percival et al., 2019). Recent studies by Matsumoto et al. (2021) revealed tuned glutamatergic inputs to DSGCs originating from T7 and T2 bipolar cells. These inputs are driven by bouton-specific cholinergic excitation, mediated by SACs in the preferred direction and GABAergic inhibition, mediated by wide-field amacrine cells in the null direction.

Asymmetries in the magnitude of inhibitory inputs onto DSGCs are mainly due to the activity of SACs, as demonstrated by paired recordings between SACs and ON-OFF DSGCs showing that inhibition is more pronounced during motion in the null direction compared to the preferred direction (Fried et al., 2002; Lee et al., 2010). Additionally, Pei et al. (2015) found that while inhibitory inputs onto DSGCs are directionally tuned to the null direction, selectively eliminating the directional tuning of inhibition in a subset of ON-OFF DSGCs did not abolish direction selectivity. This suggested that directionally tuned inhibition, while significant, may not be the sole determinant of direction selectivity. These discrepancies in the role of excitatory and inhibitory inputs underscore the need to reconcile these divergent findings.

Maintaining a consistent excitatory-inhibitory (E/I) balance has emerged as a critical factor in ensuring reliable direction selectivity across varying contrast levels. Poleg-Polsky and Diamond (2016) demonstrated that the balance between excitatory and inhibitory inputs onto DSGCs is maintained across a wide range of stimulus contrasts. This is a significant finding, as inhibition arises from feedforward inhibitory circuits where bipolar cells provide excitatory inputs to both DSGCs and SACs, with SACs subsequently inhibiting DSGCs and these types of circuits are typically known to exhibit nonlinear characteristics. Their study showed that this balance is achieved through unique compensatory mechanisms, in which bipolar cell inputs to SACs display increased contrast sensitivity. This transformation ensures that the nonlinear processing within SACs effectively adjusts the contrast sensitivity of feedforward inhibition with that of direct excitatory inputs to DSGCs, thus maintaining the balance of excitation and inhibition.

While the significance of maintaining the balance between excitation and inhibition in shaping the direction preference and spatiotemporal characteristics of DSGCs responses is widely acknowledged, the precise impact of variations in the magnitude and temporal offset of these inputs and how these inputs vary at intermediate angles between preferred and null directions remains elusive. In this study, we used synthetic excitatory and inhibitory conductances of varying amplitudes and time onsets, delivered via dynamic-clamp recordings, to dissect their contributions to direction-selective responses in RGCs. Importantly, this approach not only reproduces key features of experimentally observed direction-selective responses, but also provides a direct link between descriptive measurements and causal mechanisms, allowing us to test whether these synaptic patterns are sufficient to generate direction selectivity in RGCs. We hypothesized that changes in the amplitude and/or timing of both excitatory and inhibitory inputs will have a significant impact on the directional tuning of RGCs.

## 2 Materials and methods

### 2.1 Ethical approval

All animal procedures followed the guidelines for animal experiments issued by The University of Sydney (protocols #5085 and #661) and the Australian NHMRC Code of Practice for the Care and Use of Animals for Scientific Purposes. The experiments were carried out on both male and female adult C57Bl/6J mice, which were maintained at The University of Sydney’s animal housing facility. Mice were kept under controlled environmental conditions with a 12:12 h light/dark cycle, with constant temperature and humidity levels. Animals had unrestricted access to standard chow and water. Animals were euthanized by cervical dislocation. Efforts were made to limit the number of animals used in this study and to alleviate any potential pain or distress experienced by the animals during the procedures.

### 2.2 Tissue preparation

After euthanizing animals, eyes were rapidly enucleated and transferred to a dish containing Ames medium where the cornea and anterior part of the eye were removed and the retinae were isolated under normal light conditions. A small section of retina was isolated using a dissection microscope under white light, mounted in a recording chamber with the ganglion cell layer facing upwards and transferred to an upright Olympus microscope (BW50WI). The tissue was continuously perfused with carboxygenated Ames medium flowing at a rate of 3–5 mL/min and maintained at 35°C using an inline heater (Warner Instruments, TC-344B). Retinal tissue was visualized through the microscope under 40x magnification using infrared illumination.

### 2.3 Recordings

Whole-cell recordings were obtained from RGC somas under low ambient light conditions as previously described (Di Marco et al., 2009). Borosilicate glass electrodes (6–8 MΩ) were backfilled with a potassium gluconate based intracellular solution containing (in mM) K^+^-gluconate: 140, MgCl_2_:4.6, EGTA: 10, HEPES: 10, ATP-Na^+^: 4 and GTP-Na+: 0.4. Lucifer yellow (2%) was added to the intracellular solutions for cell identification. Initial recordings were obtained in voltage clamp mode, using an EPC8 patch clamp amplifier (HEKA Elektronik, Lambrecht, Germany). Only cells that displayed large sodium currents (>2 nA) were recorded from. Subsequently, the recording mode was switched to fast current clamp mode, with cells being held at −65 to −70 mV (corrected for liquid junction potential) to carry out dynamic clamp recordings. Recordings were excluded if access resistance, input resistance or resting membrane potential varied by more than 10–15%. All recordings were carried out in carboxygenated Ames medium. At the end of the recording, cells were visualized under fluorescent microscopy and processed for morphological identification as in Protti et al. (2014). Of the 37 RGCs in our dataset, we successfully identified 30 morphologically and classified them as monostratified types A, B, and C according to the classification by Sun et al. (2002), the identity of the remaining 7 could not be confirmed. The identified RGCs had a mean soma diameter of 15.65 ± 3.3 μm, a mean dendritic tree diameter of 277.1 ± 140.7 μm, a mean membrane resistance of 306.7 ± 288.2 Ω, and a mean firing rate of 74 ± 22 Hz, values that are consistent with those reported in the literature (Coombs et al., 2006; Kong et al., 2005; Sun et al., 2002).

### 2.4 Dynamic clamp

Dynamic clamp recordings were carried out using a field-programmable gate arrays board (FPGA, National Instruments) and custom software NeuroAkuma as described in detail in Huang et al. (2013). Recordings were performed in current clamp mode and the membrane conductance was updated every 25 μs (40 kHz). The current injected into the cell, as a function of the membrane potential, was calculated as:


$$I\,\left(t\right)=G_{e\,x\,c}\,\left(t\right)\,\left(V_{m}\,\left(t\right)-V_{e\,x\,c}\right)+G_{i\,n\,h}\,\left(t\right)\,\left(V_{m}\,\left(t\right)-V_{i\,n\,h}\right)$$

where I*_(t)_* is the total current injected at time *t*; G*_*exc*_(t)* and *G_*inh*_(t)* are the excitatory and inhibitory conductances at time *t*, and *V_*m*_(t)* is the membrane potential at time *t*. *V*_*exc*_ and *V*_*inh*_ are the excitatory (0 mV) and inhibitory (−75 mV) reversal potentials. To account for the differences in the input resistance of the cells, the magnitude of the excitatory and inhibitory conductances were scaled by a common factor whilst maintaining their ratio, in order to produce changes in membrane potential consistent with those evoked by visual stimulation. After calculation, the resulting current was fed back into the cell using the same FPGA interface operating at 40 kHz. The excitatory and inhibitory conductances are described in the next section.

### 2.5 Conductances used in dynamic clamp experiments

**FIGURE 1:**
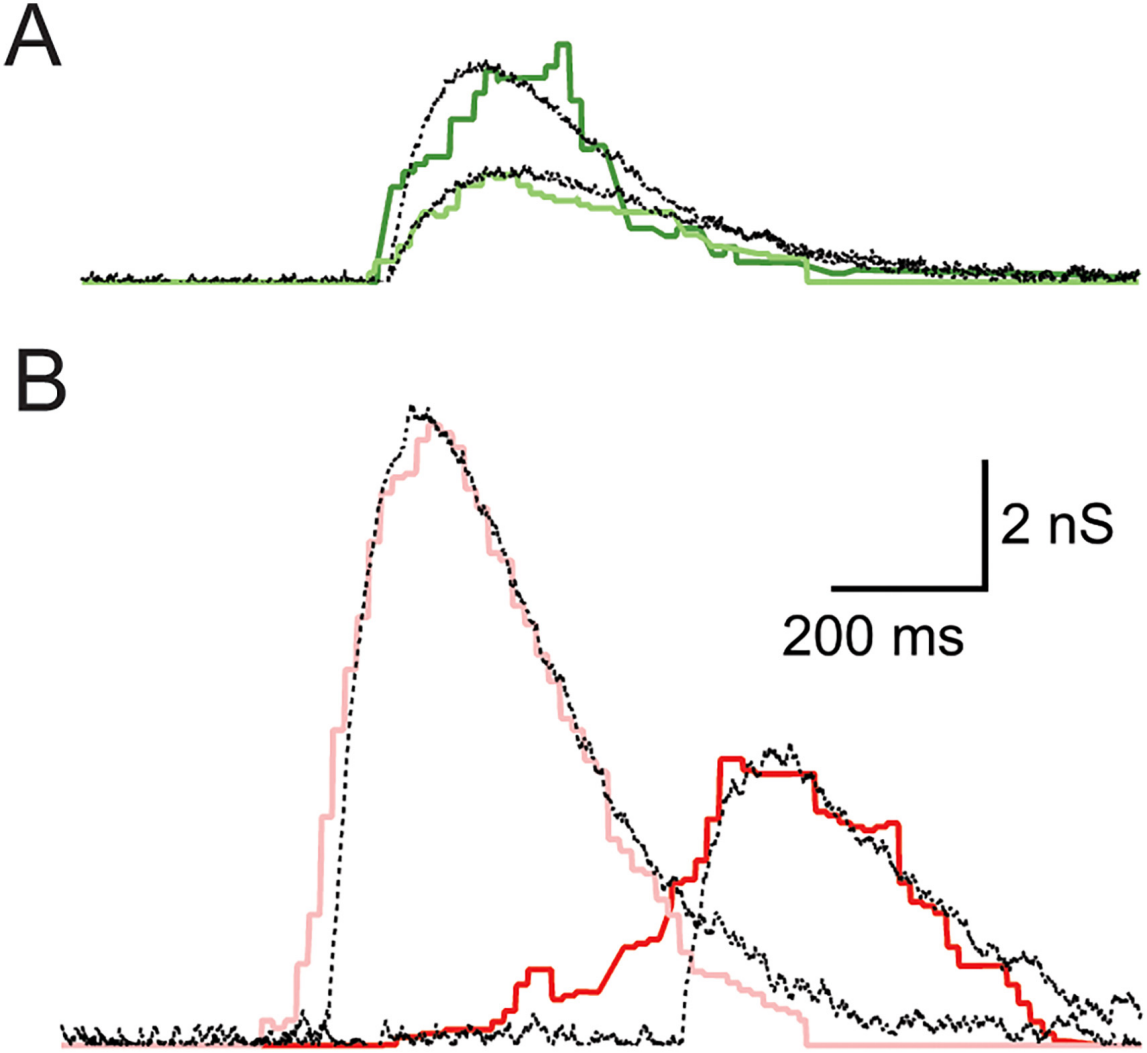
Comparison of native and synthetic synaptic conductances. **(A)** Excitatory synaptic conductance waveforms digitized from recordings by Taylor and Vaney (2002, Copyright © 2002 Society for Neuroscience; Figures 4A,B) in response to a positive contrast bar moving in the preferred (dark green) and null (light green) directions. Dotted lines represent synthetic conductances, modeled as alpha functions with a time constant (τ) of 125 ms and matching the peak amplitudes of the native conductances. **(B)** Inhibitory synaptic conductance waveforms derived from recordings by Taylor and Vaney (2002, Copyright © 2002 Society for Neuroscience; Figures 4A,B) in response to a positive contrast bar moving in the preferred (dark red) and null (pink) directions. Dotted lines show the synthetic conductances, modeled using alpha functions to match the peak amplitudes of the native conductances, with a time constant (τ) of 125 ms.

**FIGURE 2:**
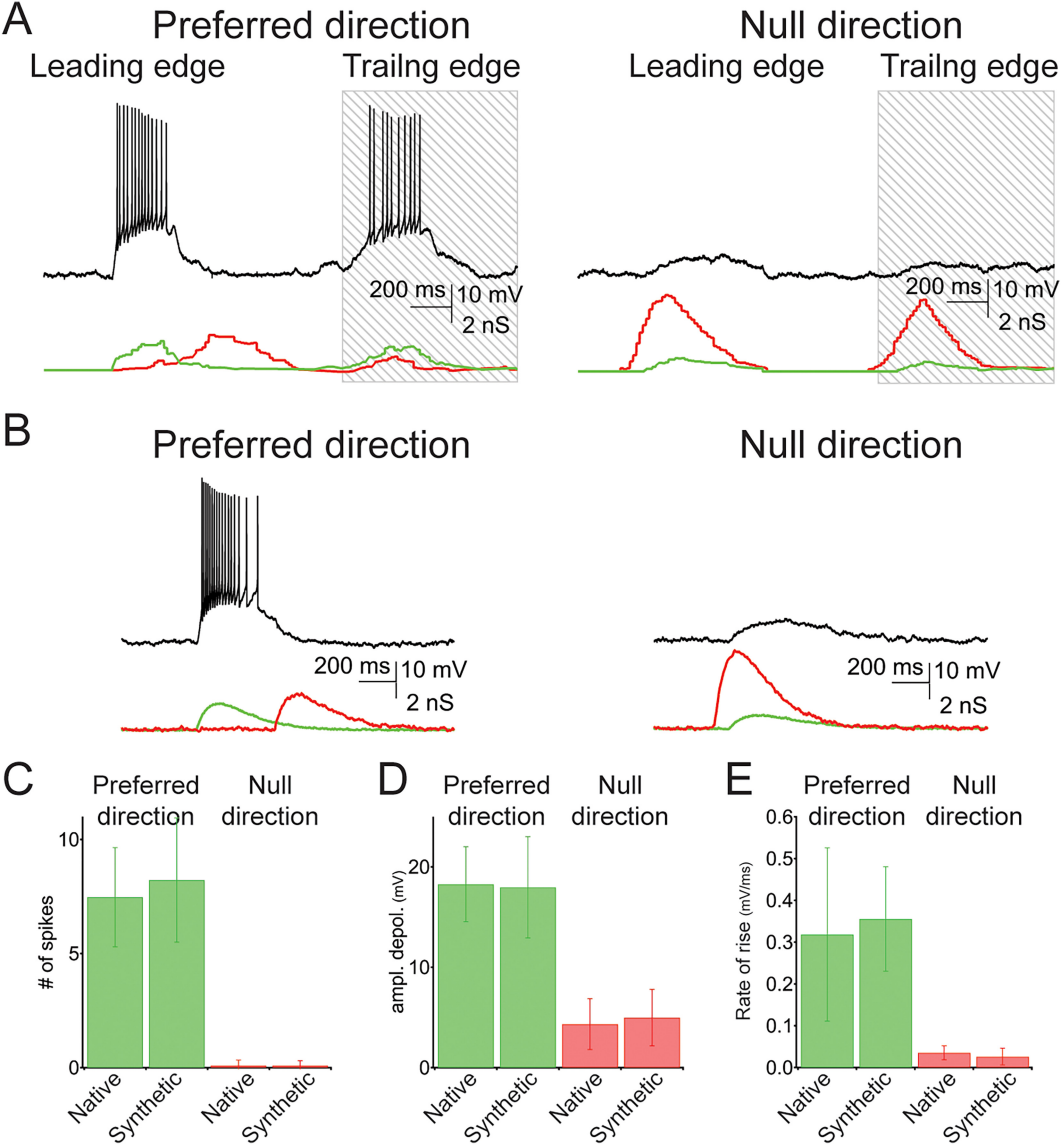
Dynamic clamp recordings with synthetic conductances replicate responses to native directionally selective conductances. **(A)** Responses to current injection based on native excitatory and inhibitory conductances for a moving bar in the preferred and null direction (left and right column, respectively). Native conductances comprise the changes in conductance to the leading and trailing edge of the bar. Membrane potential recordings (black traces) and excitatory and inhibitory conductances (green and red traces, respectively). **(B)** Responses to current injection based on synthetic excitatory and inhibitory conductances modeled on the native responses to the leading edge. Responses are shown for the preferred and null direction (left and right column respectively). Traces colors as in **(A)**. **(C–E)** Bar chart series illustrating mean spike count, mean amplitude of depolarization and mean rate of rise (slope of the depolarizing phase between 10 and 90% of response amplitude) in response to injections of native and synthetic conductances for the preferred (green) and null (red) directions. No significant differences between responses to native and synthetic conductances were detected in any of these parameters (*n* = 6 cells, *p* > 0.05).

Conductance waveforms recorded by Taylor and Vaney (2002; Figures 4A,B) in response to moving bars moving along its long axis at speeds of approximately 800–1,200 μm/s on the retina (native conductances), were digitized to obtain their time course and relative amplitudes. Although these conductances were recorded in rabbit DSGCs, here we used them as biologically grounded templates to parametrically explore how excitatory-inhibitory asymmetry shapes neuronal output, rather than to replicate species-specific circuitry. We found that the time course of both excitatory and inhibitory synaptic conductances triggered by the leading edge of a positive contrast bar can be well described by alpha functions of the form:


$$G\,\left(t\right)=G_{m\,a\,x}\,\left(t/\tau \right)\,e^{-t/\tau }$$

where *G*(*t*) is the conductance at time *t*, *G*_*max*_ is the peak conductance and τ is the time constant ranging between 120 and 130 ms (see Figure 1B). To be able to parametrically modify the amplitude and onset of the conductances, we generated waveforms (synthetic conductances) consisting of alpha functions with τ = 125 ms and their corresponding amplitudes for both preferred and null directions. Linear interpolation was used as a simple, parsimonious approach to generate conductance patterns at intermediate directions in the absence of published data. It is important to note that this model does not imply that synaptic inputs vary linearly with direction, but provides a controlled approach to systematically explore how gradual changes in excitatory-inhibitory relationships shape neuronal output. We then assumed a straightforward model in which the amplitudes and temporal offset of both excitation and inhibition were hypothesized to linearly vary from the preferred to the null direction. This allowed us to generate three intermediate directions (45, 90, and 135°) by linearly extrapolating their respective amplitudes and introducing a delay of 125 ms for the onset of inhibition for each 45° increment from the null to the preferred direction, based on a peak-to-peak difference of the inhibitory conductances of 500 ms. Figure 1 shows that the synthetic conductances closely emulate the temporal dynamics and amplitude of their native counterparts. It is important to consider, however, that a minor increase in inhibitory conductances initiates approximately 100 ms earlier in the null direction. Despite this, the model still effectively replicates its overall time course, and the absence of significant differences between responses to native and synthetic conductances supports their use (Figures 2C–E). To replicate biological signals and their distinctive variability, we added low levels of noise resembling synaptic background activity, as per the model proposed by Fellous et al. (2003), into both excitatory and inhibitory conductances. All conductance waveforms were resampled at 40 kHz for use in dynamic clamp recordings. Each combination of excitatory and inhibitory conductances was injected at least 8 times in each cell.

### 2.6 Data analyses and statistics

Data analysis was performed using custom-written routines in IGOR (Wavemetrics, Lake Oswego, OR).^1^ Spikes were identified by locating maxima derived from the smoothed first and second derivatives of the membrane potential signal. Local maxima were compared to a threshold value, and if the amplitude at that point exceeded the threshold (commonly above 0 mV), it was classified as a spike. The amplitude of the membrane potential depolarization was determined after spike removal. Spikes were removed by linearly interpolating the membrane potential signal, 4 ms before and approximately 10 ms after each spike, following (Demb et al., 1999). The amplitude of membrane potential depolarization was then calculated as the difference between the baseline membrane potential, averaged over 200 ms before the depolarizing response, and the average membrane potential across 40 ms following the peak response. The rate of rise of the depolarizing response was calculated as the slope of the rising phase between 10 and 90% of the response amplitude, defined as the change in membrane potential over this interval divided by the corresponding time. To construct polar plots, we used the measurements from 45, 90, and 135°, treating them as equivalent to their opposite counterparts at 315, 270, and 225°, respectively. This was based on their symmetrical positioning relative to key directional axes, thus ensuring congruence in the elicited conductances.

The strength of directional tuning was calculated from the responses (number of spikes or amplitude of membrane depolarization) to current injections simulating each of the 8 stimulus directions. Responses to each simulated direction were represented as vectors. The preferred direction was determined from the angle of the sum vector, obtained by summing all 8 responses, and the degree of directional selectivity was quantified by calculating the vector sum, which represented the normalized length of the resultant vector. The vector sum value can range from 0, indicating that the response magnitude was the same across all stimulus directions, to 1, indicating perfect tuning wherein a response was elicited only for a single stimulus direction (Taylor and Vaney, 2002). Directional selectivity was also assessed by calculating a direction selectivity index (DSI) for both spiking activity and amplitude of depolarization, using the equation:


$$D\,S\,I=\frac{R_{p\,r\,e\,f}-R_{n\,u\,l\,l}}{R_{p\,r\,e\,f}+R_{n\,u\,l\,l}}$$

where *R*_*pref*_ and *R*_*null*_ represent the magnitudes of the responses in the preferred and null directions, respectively.

A non-parametric Wilcoxon matched-pairs signed-rank test (hereafter Wilcoxon test) was used to compare populations of cells under different conditions. This test was chosen for its suitability in analyzing paired, non-normally distributed data. A two-tailed criterion was employed, and a P ≤ 0.05 was considered statistically significant. Comparison of these cellular responses under different conditions enabled us to establish the relationship between the integrated input a cell receives and its output response. In both the text and figures, values are presented as mean ± standard deviation (SD). Statistical analyses were conducted using Igor Pro.

## 3 Results

To examine the role of excitatory and inhibitory inputs on the generation of retinal ganglion cell direction selectivity, we made dynamic-clamp recordings in which the magnitude and timing of excitation and inhibition were parametrically modified. Our first goal was to determine if conductance waveforms, elicited by a moving bar in both preferred and null directions, could replicate the membrane responses to directionally selective visual stimuli. To do this, we injected currents based on representative excitatory and inhibitory synaptic conductances evoked by visual stimuli (see Methods) and recorded the resulting membrane potential. Figure 2A shows a representative conductance-clamp experiment using excitatory and inhibitory synaptic conductances elicited by a positive contrast bar moving in the preferred (left column) and null (right column) directions. Current injection based on conductances corresponding to the preferred direction induced a strong depolarising response and a train of action potentials for the periods corresponding to conductance changes triggered by the leading and trailing edge of the moving bar. In contrast, current injection based on conductances elicited by a bar moving in the null direction led to weaker depolarizing responses and no action potentials, validating the use of dynamic clamp experiments for replicating direction selectivity.

To systematically investigate the influence of changes in the magnitude and timing of excitatory and inhibitory inputs in the generation of direction selectivity, we modeled these synaptic conductances as alpha functions (see Methods for details) to be able to generate synthetic versions that could be parametrically modified. We then conducted conductance clamp recordings using synthetic excitatory and inhibitory waveforms with amplitude and onset matching those of native conductances (Figure 1). As shown in Figure 2B, injections based on preferred direction synthetic conductances led to strong depolarization and action potentials, while those based on null direction synthetic conductances resulted in a weaker depolarizing response and no action potentials. Figures 2C–E compare the average response strength—quantified by the number of spikes and magnitude of depolarization— and the rate of rise between native and synthetic conductances for both preferred and null directions in the same cells. We found no significant differences in these metrics (*p* > 0.15, *n* = 6 cells).

The prevailing understanding of the mechanisms generating direction selectivity involves presynaptic mechanisms that lead to stronger excitation for movement in the preferred direction and enhance inhibition for movement in the null direction combined with a temporal offset between excitation and inhibition. While changes in the magnitude and timing of these inputs for preferred and null directions are well-documented (see Matsumoto and Yonehara (2023) for recent review), to our knowledge, no study has systematically explored how the ratio and temporal offset between excitation and inhibition vary at intermediate directions and what impact they have in contributing to direction selectivity.

**FIGURE 3:**
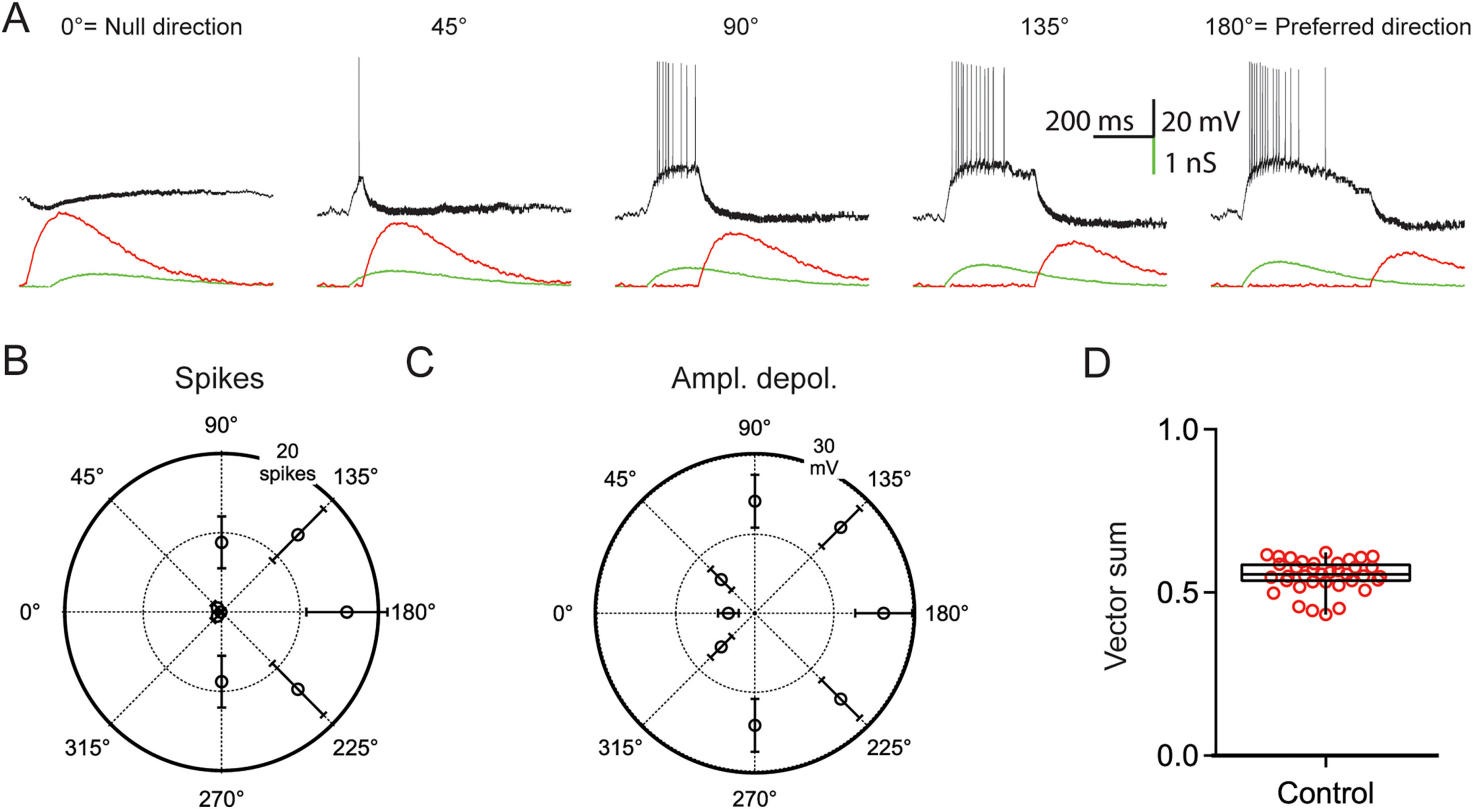
Directional selective characteristics of responses using control excitatory and inhibitory synthetic conductances. **(A)** Representative traces showing responses to current injections based on excitatory and inhibitory conductances modeled on native conductances for preferred and null direction movement and for three intermediate directions (45°, 90°, 135°). Membrane potential recordings (black traces) and excitatory and inhibitory conductances (green and red traces, respectively). **(B)** Polar plot representing mean spike count in response to current injection of conductances modeled for five different directions. Round circles on the polar plots represent the average number of spikes in each direction for the population (*n* = 37 cells). **(C)** Polar plot representing mean amplitude of membrane potential depolarization in response to current injection of conductances modeled for five different directions (*n* = 37 cells). **(D)** Box plot showing the distribution of vector sum values for all neurons injected with control excitatory and inhibitory conductances (*n* = 37 cells).

To address this gap, we used a simple model in which the magnitude and temporal offset of both excitation and inhibition are assumed to vary linearly from the preferred to the null direction. We tested the validity of the model using conductance-clamp recordings with synthetic excitatory and inhibitory conductances for the preferred (0°) and null directions (180°), as well as for three intermediate directions (45, 90, and 135°) generated by linearly extrapolating their magnitudes and delaying the onset of inhibition by 125 ms for every 45° increment from the null to the preferred direction. Figure 3A shows that the injection of currents based on these synthetic conductances resulted in spiking outputs typical of directionally selective responses, with increases in the response strength when transitioning from injections with conductances corresponding to null direction to preferred direction. Figures 3B,C display polar plots for 37 cells, showing the average spike response and amplitude of depolarization in response to injections with this set of conductances. Figure 3D shows the distribution of vector sum values centered around a mean of 0.55 ± 0.05 (*n* = 37 cells) for all neurons injected with control excitatory and inhibitory conductances, which is a comparable value to that reported in previous studies of rabbit DSGCs (0.55–0.57; see Taylor and Vaney, 2002), indicating that the model captures physiologically relevant responses. Importantly, direction-selective responses were consistently observed across all recorded cells despite variability in morphological type and intrinsic properties, indicating that the effects of conductance manipulations were robust across the population.

Seminal research by Taylor and Vaney (2002) in the rabbit retina found that the magnitude of the excitatory conductance was greater in the preferred direction compared to the null direction. In contrast, direct measurements of glutamate release from bipolar cells in the mouse retina found that excitatory output is not directionally selective, suggesting the possibility that these differences may actually stem from errors in quantifying excitation using voltage-clamp techniques during null-direction inhibition (Park et al., 2014). A subsequent analysis in rabbit retina directly tested this hypothesis and concluded that directional excitation in rabbit DSGCs could not be accounted for by voltage-clamp errors (Percival et al., 2019). Moreover, recent studies in mouse have found that bipolar cell output is directionally selective (Matsumoto et al., 2021; Yonehara et al., 2013). The direction-selectivity of excitation is relatively weak (Fried et al., 2002; Taylor and Vaney, 2002), however, it is the major driver of spiking and since the spike threshold amplifies the strength of directional tuning (Oesch et al., 2005; Sivyer and Williams, 2013), even small directional-differences in the excitatory conductance could produce significant effects.

**FIGURE 4:**
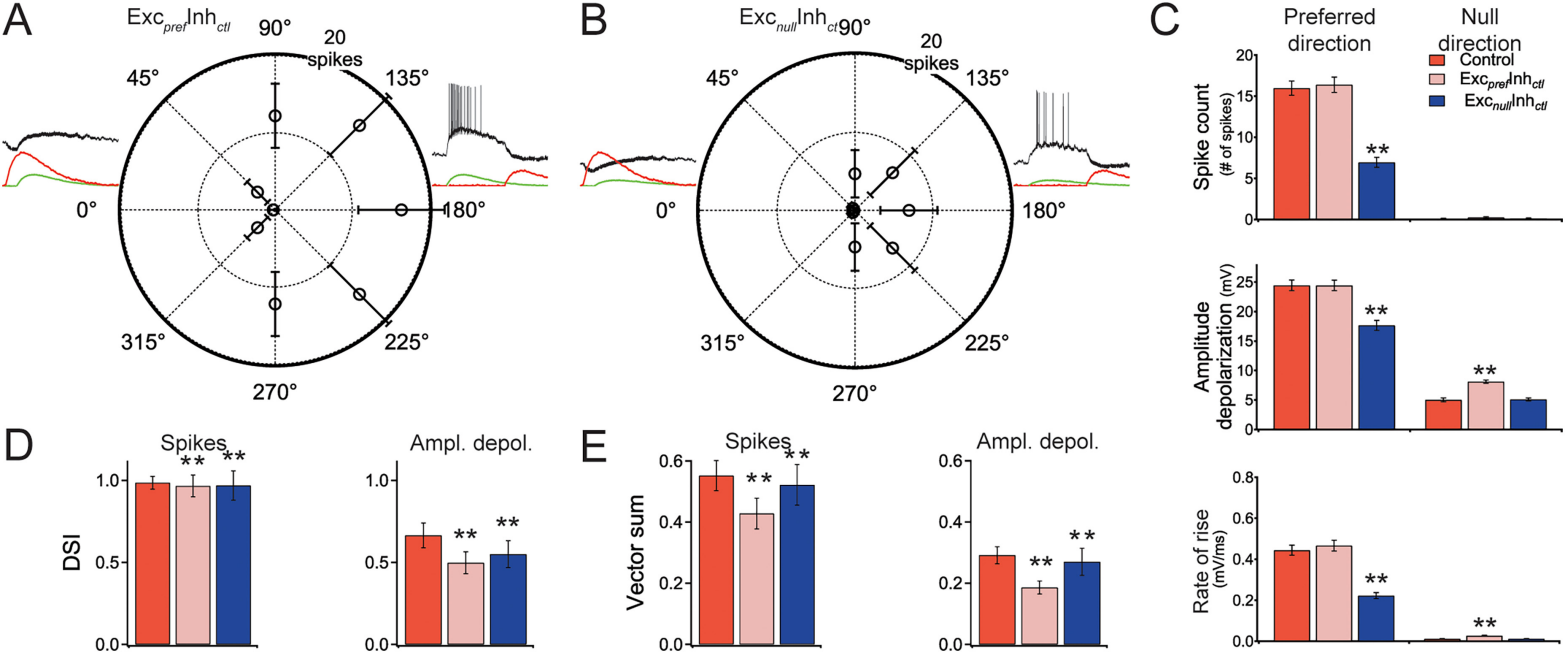
Removal of direction tuning of excitatory inputs leads to weaker direction selectivity. **(A)** Polar plot representing mean spike count in response to current injection of conductances modeled for five different directions for current injections based on preferred excitatory and control inhibitory conductances (*n* = 37 cells). **(B)** Polar plot representing mean spike count in response to current injection of conductances modeled for five different directions for current injections based on null excitatory and control inhibitory conductances (*n* = 37 cells). Representative traces showing responses to current injections based on excitatory and inhibitory conductances for preferred and null direction movement for conditions shown in polar plots A and B. Membrane potential recordings (black traces) and excitatory and inhibitory conductances (green and red traces, respectively). **(C)** Bar charts representing mean spike count, amplitude of depolarization and rate of rise of responses to current injections with three distinctive combinations of conductances: control excitatory and control inhibitory conductances, preferred excitatory and control inhibitory as well as null excitatory and control inhibitory conductances in the preferred and null directions. **(D)** Strength of directional selectivity assessed by the DSI for spike count and amplitude of depolarization across the three combinations of conductances. **(E)** Vector sum for spike count and amplitude of depolarization measured in response to the three combinations of conductances. **p < 0.01.

To assess the impact of changes in the amplitude of the excitatory conductance on direction selectivity, we measured responses with the excitatory conductance set to either its preferred or null direction value, in the presence of the same control inhibitory conductances. First, we set the excitatory conductance to its preferred direction value and co-injected it with synthetic inhibitory conductances, simulating five different directions ranging from null to preferred, based on our model. Figure 4A shows the polar plot of average spike responses for 37 cells. Elimination of the direction-dependent reduction in excitatory conductance magnitude from preferred to null direction did not significantly modify the strength of the spike response quantified as the mean spike count (16.39 ± 0.9 spikes vs. 15.98 ± 0.9 spikes in control conditions, *n* = 37 cells, *p* = 0.5, Wilcoxon test). However, it did lead to a significant reduction in direction selectivity, as indicated by both the DSI and the vector sum (Figures 4D,E, *n* = 37 cells, *p* < 0.01) for spike responses. The strength of the spike response in the null direction was not significantly altered. The amplitude of the depolarizing response in the preferred direction remained largely unaffected by the removal of direction-dependent changes in excitatory inputs (Figure 4C). In the null direction, however, there was a significant increase in the amplitude of the depolarizing response (8.1 ± 1.6 mV vs. 5.03 ± 1.9 mV in control conditions, *n* = 37 cells, *p* < 0.01; Figure 4C). Both the DSI and the vector sum of the depolarizing response, however, exhibited a significant reduction (Figures 4D,E, *n* = 37 cells, *p* < 0.01). The rate of rise of the depolarizing response in the preferred direction remained unchanged while in the null direction it was significantly increased to 0.03 ± 0.01 mV/ms from 0.01 ± 0.005 mV/ms in control conditions (*n* = 37 cells, *p* < 0.01; Figure 4C). These findings suggest that the reduction in the magnitude of the excitatory input in the null direction may contribute to enhance the degree of direction selectivity.

Next we fixed the excitatory conductance at its null direction value and measured responses over the same range of inhibitory conductances. Under these conditions, the excitation-to-inhibition ratio is smaller for all directions except the null direction. Figure 4B shows the polar plot of the average spike responses for 37 cells. Maintaining excitation at its null direction value led to a significant reduction to 6.9 ± 3.6 spikes in maximum spike output compared to control conditions (*n* = 37 cells, *p* < 0.001, Figure 4C) and led to a significant reduction in direction selectivity, as indicated by both the DSI and the vector sum (Figures 4D,E, *n* = 37 cells, *p* < 0.001). The amplitude of the depolarising response in the preferred direction was significantly reduced to 17.66 ± 5 mV, from 24.4 ± 5.4 mV in control conditions, by the removal of directional-dependent changes in excitatory inputs (*p* = 0.001). Additionally, the DSI and the vector sum calculated from the depolarising response exhibited a significant reduction (Figures 4D,E). The rate of rise of the peak depolarising response in the preferred direction was also significantly reduced (Figure 4C, 0.2 ± 0.08 mV/ms vs. 0.43 ± 0.14 mV/ms, *n* = 37 cells, *p* = 0.001). These findings suggest that the reduction in E/I ratio and the absence of asymmetry in the magnitude of the excitatory inputs leads to a weakened response, rendering ganglion cells less directionally selective and this can be attributed to a decrease in both the amplitude and rate of rise of the depolarising response.

**FIGURE 5:**
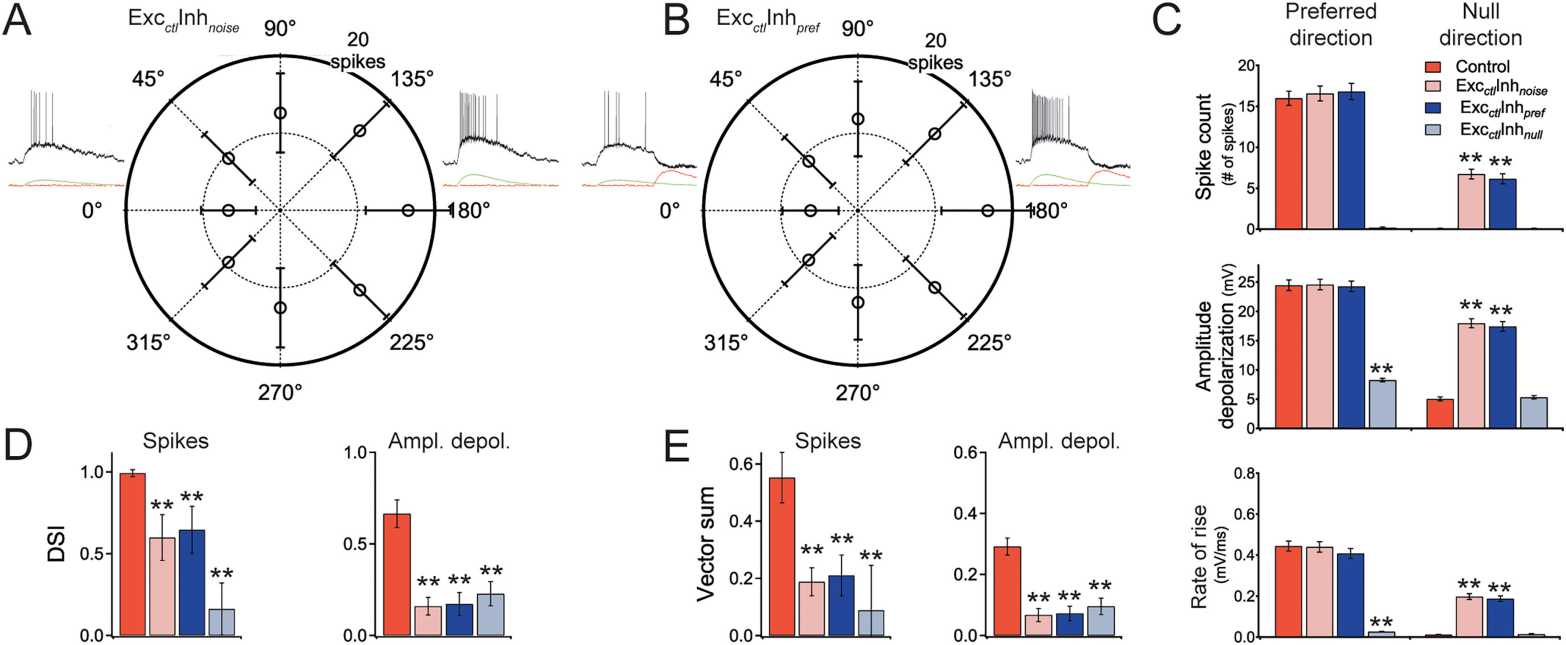
Changes in the magnitude of inhibitory inputs leads to weaker direction selectivity. **(A)** Polar plot representing mean spike count in response to current injection of conductances modeled for 5 different directions for current injections based on control excitatory and baseline noise inhibitory conductances. **(B)** Polar plot representing mean amplitude of membrane potential depolarization in response to current injection of conductances modeled for five different directions for current injections based on control excitatory and baseline preferred inhibitory conductances. Round circles on the polar plots represent the average number of spikes in each direction for the population (*n* = 37 cells). Representative traces showing responses to current injections based on excitatory and inhibitory conductances for preferred and null direction movement for conditions shown in polar plots A and B. Membrane potential recordings (black traces) and excitatory and inhibitory conductances (green and red traces, respectively). **(C)** Bar charts representing mean spike count, amplitude of depolarization and rate of rise of responses to current injections with four distinctive combinations of conductances: control excitatory and control inhibitory, control excitatory and noisy inhibitory, control excitatory and preferred inhibitory as well as control excitatory and null inhibitory conductances in the preferred and null directions. **(D)** Strength of directional selectivity assessed by the DSI for spike count and amplitude of depolarization across the four combinations of conductances. Vector sum values for spike count and amplitude of depolarization measured in response to the four combinations of conductances. **(E)** Vector sum for spike count and amplitude of depolarization measured in response to the four combinations of conductances. **p < 0.01.

To assess the degree to which direction-selective responses are shaped by variations in the magnitude of excitation between preferred and null directions, we conducted experiments in the absence of stimulus evoked changes in the magnitude and timing of inhibitory inputs. This condition also provides insights into the contribution of inhibition to the generation of direction selectivity. In this scenario, the excitatory conductances were varied as in control conditions and the inhibitory conductances consisted of simulated noisy synaptic input (see Materials and methods for details). Figure 5A displays polar plots for spike responses for the population of 37 cells recorded. Eliminating the stimulus-evoked increases in the magnitude of inhibition did not affect the maximum spike output compared to control conditions (*p* > 0.4, Figure 5C). However, it led to a significant increase in the null direction responses to 6.7 ± 3.5 spikes from 0.1 ± 0.4 spikes in control conditions (*p* < 0.001, Figure 5C). The significant decrease in both the DSI and vector sum indicates a reduction in direction selectivity and confirms that stimulus-evoked increases in inhibition are critical for the generation of robust directional tuning (Figures 5D,E). The mean amplitude of the depolarizing response in the preferred direction remained unchanged (24.5 ± 5.5 mV vs. 24.4 ± 5.4 mV) but there was a significant loss of its directionality as indicated by a decrease in the DSI (*n* = 37 cells, *p* < 0.001, Figure 5D). The rate of rise of the depolarizing response for preferred direction remained almost unchanged (0.45 ± 0.15 mV/ms vs. 0.43 ± 0.14 mV/ms in control conditions, *n* = 37 cells; Figure 5C). The removal of stimulus-induced inhibition in the null direction resulted in a significant increase in both the amplitude and rate of rise of the depolarizing response (*p* > 0.29; Figure 5C).

Having confirmed the pivotal role of inhibitory inputs, we further explored how changes in the strength and timing of inhibitory inputs contribute to directional tuning, by employing two distinct protocols. In the first, we co-injected control excitatory conductances along with inhibitory conductances whose magnitude was set to match that of the preferred direction and their timing adjusted to align with that of each specific direction under investigation. Our hypothesis posits that the absence of an increase in inhibitory magnitude from the preferred to null direction will result in reduced direction selectivity, characterized by a decrease in directional tuning. In the second protocol, we used a similar approach using inhibitory conductances with magnitudes corresponding to the null direction. Our hypothesis anticipates that by keeping the inhibitory conductance magnitude constant from the null to preferred direction, we will observe a reduction or absence of the response gradient characteristic of direction selective responses.

For the first protocol, the mean spike response, amplitude of depolarization, and rate of rise in the preferred direction were similar to those in control conditions. However, all these measurements showed significant increases in the null direction (Figure 5C). Figures 5D,E reveal that the lack of increase in inhibitory input from preferred to null direction leads to a significant loss of direction selectivity of both the spike response and the amplitude of the depolarization, as evidenced by both the DSI and the vector sum.

For the second protocol, co-injecting inhibitory conductances from the null direction along with control excitatory conductances nearly eliminated spike responses in all five directions for all cells tested (*p* < 0.001; Figure 5C). The mean amplitude and rate of rise of the depolarizing response in the preferred direction were also significantly reduced (Figure 5C). As expected, neither the mean amplitude of the depolarizing response nor the rate of rise in the null direction were significantly affected (Figure 5C). Figures 5D,E reveal a requirement for a marked reduction in the strength of the inhibitory input from null to preferred direction for the emergence of direction selectivity. Under these conditions, both DSI and the vector sum for the spike responses and the amplitude of depolarization were significantly affected. Taken together, these findings highlight the pivotal role of the increase in the magnitude of the inhibitory inputs when transitioning from preferred to null direction for the emergence of sharp directional tuning.

**FIGURE 6:**
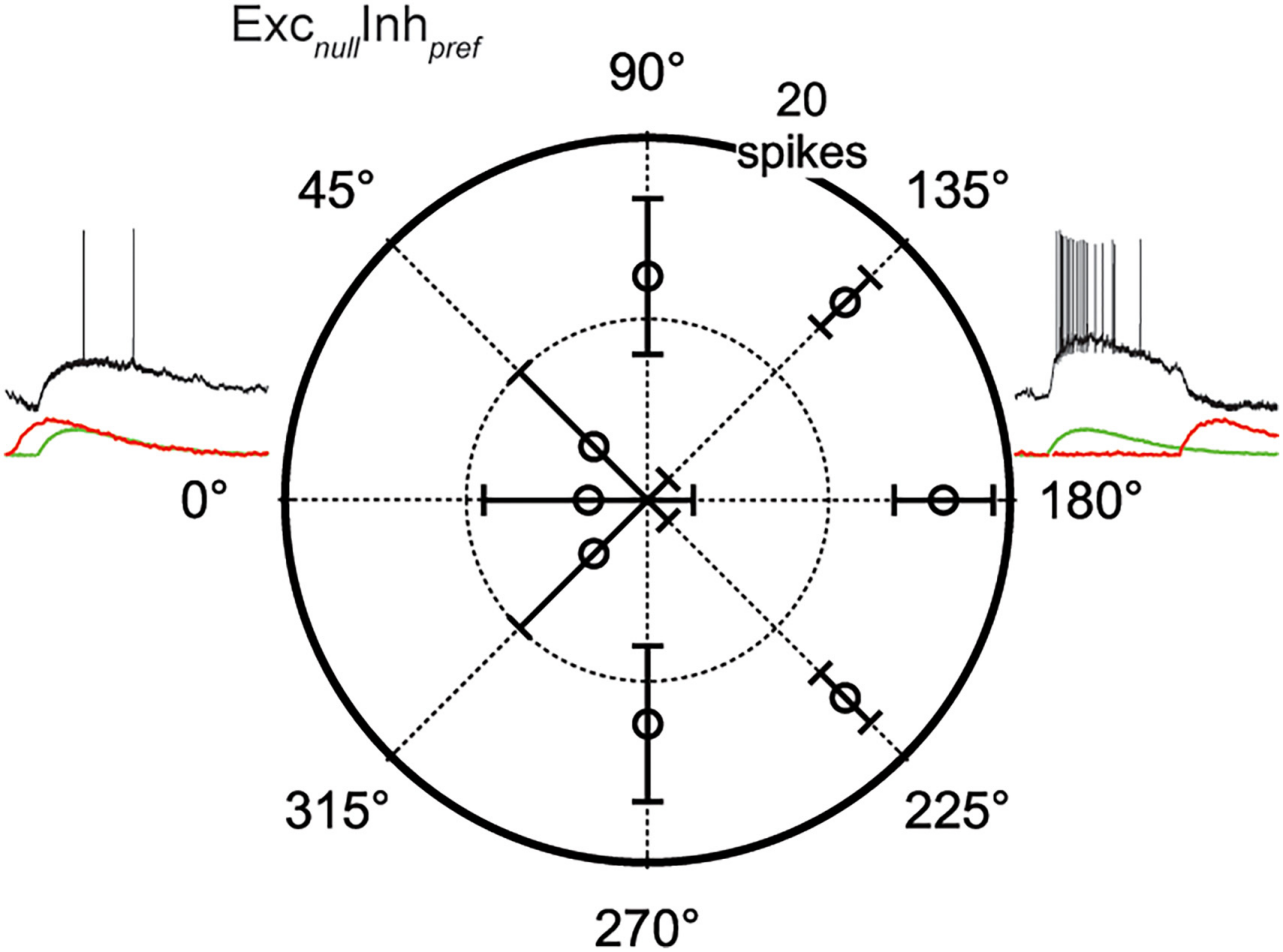
Temporal offset of inhibition with fixed conductance amplitudes induces direction selectivity. Polar plot representing mean spike count in response to current injection of conductances modeled for 5 different directions for current injections based on the excitatory conductance fixed at its null direction value and the inhibitory conductance fixed at its preferred direction value whilst varying the temporal offset to simulate different motion directions. Round circles represent the average number of spikes in each direction for the population (*n* = 37 cells). Representative traces showing responses to current injections based on excitatory and inhibitory conductances for preferred and null direction movement for conditions shown in polar plots A and B. Membrane potential recordings (black traces) and excitatory and inhibitory conductances (green and red traces, respectively).

To gain further insight into the role of the temporal offset between excitation and inhibition in direction selectivity, we conducted experiments where we fixed the amplitude of the excitatory conductance at its null direction value and the inhibitory conductance at its preferred direction value and varied the temporal offset of the inhibitory conductance to simulate different motion directions while keeping the conductance magnitudes constant. This approach allowed us to isolate the effect of inhibitory timing on neuronal output. Figure 6 shows a polar plot for the mean spike count under these conditions. Despite the fixed amplitudes, responses exhibited directional tuning driven by the timing of inhibition relative to excitation. Neuronal responses were strongly reduced when inhibition preceded excitation (null direction) and enhanced when inhibition was delayed relative to excitation (preferred direction). Quantitative analysis yielded a DSI of 0.69 ± 0.24 and a vector sum value of 0.35 ± 0.09 (*n* = 37 cells), indicating a moderate level of direction selectivity achieved solely through the temporal offset between excitation and inhibition. The mean depolarizing response amplitude in the preferred direction remained unchanged under this condition (24.4 ± 5.2 mV vs. 24.4 ± 5.4 mV; *n* = 37 cells). In contrast, the mean amplitude of the depolarizing response in the null direction significantly increased (15.5 ± 2.5 mV vs. 5 ± 1.9 mV, *n* = 37 cells). This increase led to a significant reduction in directionality, as reflected by a decrease in the DSI from 0.66 ± 0.08 to 0.22 ± 0.07 (*n* = 37 cells, *p* < 0.001). The rate of rise of the depolarizing response in the preferred direction showed no change (0.43 ± 0.16 ms vs. 0.43 ± 0.14 mV/ms, *n* = 37 cells) but was significantly increased in the null direction (0.13 ± 0.05 ms vs. 0.01 ± 0.005 ms, *n* = 37 cells). These findings further highlight the critical role of temporal dynamics in shaping direction selectivity.

## 4 Discussion

Our results unequivocally confirm that changes in the magnitude of both inhibitory and excitatory inputs as well as their temporal offset are crucial for the generation of DS. Our findings support a model in which excitatory inputs exhibit direction selectivity, as removal of direction-dependent changes in excitatory conductance magnitude significantly reduced direction selectivity. Stronger excitation in the null direction caused an increase in the amplitude and a shorter rate of rise of the depolarizing response, consequently leading to increased spiking output in the null direction and to a loss of direction selectivity. This observation can be explained by the noted increase in the amplitude and the faster rate of rise of the depolarizing response in the null direction, despite no significant changes in these parameters and in the spike response in the preferred direction. Maintaining excitatory inputs at their null direction value resulted in a marked reduction in maximum spike output and a significant decrease in direction selectivity compared to control conditions. This decrease in response strength primarily originates from a reduction in the amplitude and a slower rate of rise of the depolarizing response in the preferred direction. Furthermore, the balance of excitation to inhibition at preferred, null, and intermediate directions emerges as a critical factor for robust directional tuning.

Recent electrophysiological and imaging studies on the directional tuning of excitatory inputs onto DSGCs have yielded conflicting results, with some reports showing stronger excitatory inputs from the preferred side (Hanson et al., 2023; Lee et al., 2010; Pei et al., 2015; Percival et al., 2019), while others reporting comparable magnitudes to null direction inputs (Hanson et al., 2019; Park et al., 2014; Sethuramanujam et al., 2017). More recently, acetylcholine imaging studies reported symmetrical distribution of cholinergic excitation at the global level (Sethuramanujam et al., 2021). However, this work primarily focused on select ON-OFF DSGC types, leaving open the possibility that other ON-OFF and ON DSGC subtypes may exhibit biased distributions of preferred cholinergic synapses. Intriguingly, they also provided compelling evidence for direction tuning in cholinergic inputs onto DSGC dendrites at the local scale, although lacking global order as they did not exhibit a clear correlation with the DSGC’s preferred-null axis, challenging the notion of a directional bias in cholinergic excitation towards the DSGC’s preferred direction. The conductance templates used in our study reflect a specific range of motion processing. Direction-selective mechanisms can vary with both stimulus speed and DSGC subtype. For example, Matsumoto et al. (2019) showed that ON DSGCs achieve direction selectivity at slow speeds through the spatiotemporal summation of non-direction-selective excitatory inputs. This mechanism differs from classical models of ON-OFF DSGCs, which rely on asymmetric inhibition and excitation-inhibition timing difference. Thus, our findings here may reflect mechanisms operating in ON-OFF DSGCs or at intermediate-to-higher speeds, and their generalization to other regimes remains to be determined. These findings collectively highlight the complex nature of directional tuning in excitatory inputs onto DSGCs, shedding light on the intricate interplay of factors governing this phenomenon. Nonetheless, our results emphasize the significance of excitation input asymmetry and a precise excitation-to-inhibition balance in shaping direction selectivity in ON-OFF DSGCs and align with substantial evidence supporting directional tuning of excitation (Fried et al., 2002; Percival et al., 2019; Taylor and Vaney, 2002; Weng et al., 2005). Future research should explore the impact of different excitatory sources (glutamatergic and cholinergic) on shaping direction selectivity, as Hanson et al. (2019) demonstrated that cholinergic inputs are crucial for early spike initiation, while glutamatergic inputs modulate later response phases, particularly under varying contrast conditions where NMDA receptors amplify cholinergic inputs in low contrast conditions (Sethuramanujam et al., 2016).

The removal of the stimulus-induced increase in inhibition had no impact on the maximum spike output compared to control conditions. However, this manipulation significantly increased responses in the null direction, leading to a significant loss of direction selectivity. This reduction in direction selectivity was caused by an increase in response strength in the null direction for all parameters measured. These findings underscore the critical role of inhibition in shaping direction selectivity. Specifically, our results show that the increase in the magnitude of the inhibitory input in the null direction is critical for the establishment of robust directional tuning. The removal of the increase in inhibition in the null direction led to a marked reduction in direction selectivity, as evidenced by significant increases in mean spike response, amplitude of depolarization, and rate of rise. This is consistent with the findings of Pei et al. (2015), who reported that direction selectivity persists but is reduced in a subset of ON-OFF DSGCs when directionally tuned inhibition is abolished. Our results show a reduction in vector sum values similar in magnitude to that reported by Pei et al. (2015). Additionally, the reduction in the magnitude of the inhibitory input in the preferred direction is equally crucial, as maintaining inhibition at its null direction value resulted in a complete absence of spikes when co-injected with excitatory conductance of the preferred direction. Our model, featuring linear modulation of the amplitude of excitatory and inhibitory inputs from the preferred to null direction, aligns well with the current understanding of the connectivity pattern in the DS circuit, where asymmetric connections between SACs and DSGCs underlie the computation of direction selectivity. Although SAC synapses onto DSGCs span their entire dendritic field, they were found to be predominantly present on the null side of DSGCs (∼90% vs. 10%; Briggman et al., 2011). In this scenario, stimuli transitioning from the preferred to null direction will elicit increasing inhibitory inputs from SACs oriented along the null direction—a phenomenon likely shaped by SAC dendritic spatial arrangements. At intermediate stimulus angles, multiple SAC dendrites, acting as localized computational units for motion detection for that particular angle, will engage. As a result, the magnitude of signals generated by these dendrites will be proportional to the degree of stimulus alignment. A similar mechanism, involving SACs or other inhibitory interneurons, may also contribute to the suppression of excitatory inputs as the stimulus transitions from the preferred to the null direction, further tuning excitation (Fried et al., 2002; Fried et al., 2005; Zhou and Lee, 2008).

Changes in the magnitude of the rate of rise correlated with changes in the amplitude of the depolarizing response, reflecting the integration of excitatory and inhibitory inputs. Typically, reductions in the rate of rise of depolarizing responses were observed in preferred direction responses when both excitation and inhibition were set to their null direction values. Conversely, increases in the rate of rise occurred in null direction responses when excitation was at the preferred direction value, or when inhibition was removed or set at the preferred direction value. Functionally, slower rates of rise may lead to the inactivation of voltage-gated sodium channels during prolonged depolarizations, thus reducing neuronal excitability (Carter et al., 2012). As direction selectivity depends on robust activation in response to preferred-direction stimuli and minimal activation in the null direction, these changes in rate of rise are consistent with the changes observed in direction selectivity parameters.

Our results demonstrate that varying the temporal offset of inhibition relative to excitation can induce direction selectivity even when the amplitude of excitation and inhibition are held constant. This highlights the critical role of inhibitory timing in shaping neuronal responses and its significant contribution to directional tuning. These results emphasize the importance of temporal dynamics in the computation of direction selectivity. Furthermore, our data show that amplitude and timing work synergistically to enhance direction selectivity. While moderate selectivity can be achieved through adjustments to the temporal offset of inhibition alone, the highest levels of direction selectivity occur when both synaptic amplitudes and timing are varied together.

While previous studies have identified temporal offsets between excitation and inhibition as a key determinant of direction selectivity (Fried et al., 2005; Taylor and Vaney, 2002), our results extend this model by demonstrating how these temporal relationships interact with systematic changes in input magnitude to shape directional tuning across the full range of stimulus directions. In contrast to earlier work, which mainly inferred these mechanisms from physiological recordings, our approach provides direct, independent control of excitatory and inhibitory conductances. This allows dissociation of the contributions of amplitude and timing, demonstrating that although temporal offsets alone can generate moderate direction selectivity, robust tuning emerges from the coordinated modulation of both parameters. These findings therefore provide experimental evidence that links previously proposed mechanisms to a unifying transformation from synaptic input asymmetry to directional output.

A potential limitation of our approach is that conductance templates derived from rabbit DSGCs were applied to mouse RGCs with diverse intrinsic properties and cell types. Although species-and cell-type-specific differences in membrane capacitance, resistance and spike-generation mechanisms can shape synaptic integration, our results indicate that the transformation from excitatory-inhibitory asymmetry to directional output is robust to this variability. Our approach to investigate the mechanisms generating direction selectivity through dynamic-clamp recordings with synthetic conductances, provides a controlled platform to study the effects of the balance of excitation and inhibition on membrane potential and spike output. While we did not directly record from DSGCs and injected conductances at the soma, the consistent reproduction of typical directional selectivity behavior suggests that this technique can offer a unique opportunity to investigate the impact of isolated conductances on the DS of RGCs, yielding valuable insights into the underlying mechanisms of DSGC tuning. Nevertheless, it is important to note that our study exclusively examined the impact of changes in excitatory and inhibitory inputs on DS by somatic injection of currents modeled in conductances measured at the soma, thus bypassing the contribution of dendritic integration to directional tuning. The use of somatically recorded conductances in dynamic clamp experiments to investigate their role in spiking output has been validated by previous research (Cafaro and Rieke, 2010; Murphy and Rieke, 2006; Summers and Feller, 2022). Cafaro and Rieke (2010) demonstrated that injecting synthetic conductances via dynamic clamp successfully captures key aspects of light-evoked responses in ON-OFF mouse DSGCs. Their study showed that noise correlations between excitatory and inhibitory synaptic inputs play a critical role in enhancing direction selectivity by improving the temporal fidelity of the spike responses. They acknowledged that somatic injections of synaptic inputs might not fully mimic dendritic voltage-activated conductances; however, they concluded that for these cells, somatic injection successfully recapitulated stimulus selectivity, response kinetics and spike response variability, even though peak firing rates fell short of those observed with light inputs. Summers and Feller (2022) demonstrated that by integrating conductances estimated from voltage-clamp experiments in ON-OFF DSGC, they could successfully recapitulate the amplitude and directional selectivity observed in current clamp measurements. Similarly, Murphy and Rieke (2006), carried out conductance clamp experiments using somatic injections into large ON and OFF RGCs and highlighted the critical role of the conductances measured at the soma in governing the cell’s output. A recent study by Wienbar and Schwartz (2022) used dynamic clamp experiments to demonstrate that differences in spike generation mechanisms and susceptibility to depolarization block were critical in shaping the contrast responses of two RGC types, the bursty Suppressed-by-Contrast (bSbC) and OFF sustained alpha (OFFsA) cells, when exposed to the same synaptic inputs. In contrast, we found that despite variability in RGC types and their intrinsic properties, all recorded cells exhibited typical directionally selective behavior in response to injections of directionally selective conductances. This suggests that the specific combination of conductances used in our dynamic clamp experiments may have overridden the impact of intrinsic properties, producing a uniform response across cell types. It is possible that the directional conductances used in our experiments engaged active properties in a way that minimized the impact of variability in intrinsic properties, raising the possibility that some patterns of conductances can reveal whilst others mask the impact of intrinsic properties. Future work using dynamic-clamp recordings could explore how changes in conductances, such as changes in NaV channel density or axon initial segment length, impact directional selectivity in different RGC types. Nevertheless, similar to the findings of Wienbar and Schwartz, who showed that active dendritic processing was not required to reproduce the distinct outputs of bSbC and OFFsA RGCs, our results demonstrate that RGCs can still exhibit robust directionally selective behavior in the absence of modeled active dendritic properties. This suggests that in both studies, the conductances used in dynamic clamp experiments were sufficient to capture the key response features of the RGCs, despite not accounting for dendritic spikes. An additional consideration is that the conductance templates used here were derived from responses to stimuli moving at speeds of approximately 800–1,200 μm/s. As previous studies have shown that different mechanisms may contribute to direction selectivity at different stimulus speeds (Matsumoto et al., 2019; Summers and Feller, 2022), the extent to which the principles identified here generalize across speed ranges remains to be determined.

Another interesting aspect to consider in our approach is the known presence of significant nonlinearities in the circuitry governing direction-selective responses in ON-OFF DSGCs. These nonlinearities encompass both presynaptic and postsynaptic mechanisms, such as threshold activation of voltage-gated processes, dendritic spike activation, NMDA receptor activation and nonlinearities in the SAC responses (Hausselt et al., 2007; Huang et al., 2022; Jain et al., 2020; Koren et al., 2017; Oesch et al., 2005; Vaney et al., 2012). DSGCs have been shown to generate dendritic spikes, suggesting that direction-selective subunits can operate independently through this mechanism (Oesch et al., 2005). Furthermore, Sivyer and Williams (2013) demonstrated that local direction-selective dendritic responses are further enhanced by dendritic spikes that propagate to the soma, amplifying spiking direction selectivity. Thus, it is assumed that dendritic spike generation ensures faithful transmission of responses from direction-selective subunits to the soma, regardless of their origin within the dendritic tree. The dendritic spike propagation model emphasizes localized interactions between excitation and inhibition, with dendritic spike initiation as a critical nonlinearity (Schachter et al., 2010). In addition, shunting inhibition and compartmentalization of synaptically-evoked calcium signals also introduce nonlinearity, potentially promoting independence among dendritic regions in terms of their directional tuning properties (Jain et al., 2020).

The aforementioned differences between our approach and physiological recordings from ON-OFF DSGCs are likely to explain some disparities in parameters such as response strength, as measured by spike count. In fact, Oesch et al. (2005) found that light-evoked postsynaptic potentials produced more spikes and higher spike rates at similar or less depolarized levels than responses to direct current injection, which mimicked the light-evoked PSPs in the same DSGCs. Furthermore, dynamic clamp recordings with ON-OFF DSGCs light-evoked conductances in ON-OFF DSGCs produced weaker spiking output compared to visual-evoked responses, highlighting the critical role of dendritic integration but still capturing key directional tuning features (Summers and Feller, 2022). Our findings, despite similar differences in response strength to those previously observed, demonstrate that a simplified model, featuring linear changes in excitatory and inhibitory input magnitudes combined with temporal offsetting and without explicit consideration of nonlinearities, effectively reproduces robust direction selectivity. It is thus possible that dendritic spikes amplify or speed up direction selective signals, but their absence does not seem to preclude achieving direction selectivity. Our results shed light on the essential changes in the amplitude and timing of both excitation and inhibition required for the emergence of directionally selective responses, as well as on their constraints. These findings also offer new perspectives on potential synaptic mechanisms that may contribute to the generation of direction selectivity, particularly at intermediate directions, involving linear changes in the magnitude of excitatory and inhibitory inputs combined with temporal offsetting.

Together, our results highlight the multifaceted role played by the directional tuning of both excitatory and inhibitory inputs in the emergence of direction selectivity in RGCs, reinforcing previous findings that emphasize the contribution of various mechanisms to this phenomenon. Moreover, these findings underscore a seemingly convergence in the functions attributed to both excitatory and inhibitory network mechanisms in the generation of direction selectivity, thus contributing to a partial reconciliation of divergent perspectives and enhancing our understanding of the fundamental mechanisms underpinning direction selectivity.

## Data Availability

The raw data supporting the conclusions of this article will be made available by the authors, without undue reservation.

## Footnotes

1
https://www.wavemetrics.com/products/igorpro
